# Diaqua­bis[2-(2-pyridylmeth­oxy)pyrazine-κ*N* ^4^]bis­(thio­cyanato-κ*N*)cobalt(II)

**DOI:** 10.1107/S1600536808030882

**Published:** 2008-09-30

**Authors:** Zhong Nian Yang

**Affiliations:** a Department of Chemistry and Chemical Engineering, Binzhou University, Binzhou 256603, People’s Republic of China

## Abstract

In the title complex, [Co(NCS)_2_(C_10_H_9_N_3_O)_2_(H_2_O)_2_], the Co^II^ ion is located on a crystallographic twofold rotation axis and is in a slightly distorted octa­hedral CoN_4_O_2_ coordination environment. The dihedral angle between the pyridine and pyrazine rings is 85.86 (10)°. In the crystal structure, inter­molecular O—H⋯N and O—H⋯S hydrogen bonds link complex mol­ecules into a three-dimensional network.

## Related literature

For the isostructural Mn complex, see: Li (2007 ▶). For a related structure, see: Zhao *et al.* (2007 ▶).
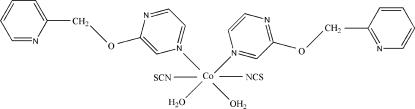


## Experimental

### 

#### Crystal data

[Co(NCS)_2_(C_10_H_9_N_3_O)_2_(H_2_O)_2_]
*M* *_r_* = 585.53
Monoclinic, 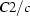

*a* = 19.954 (4) Å
*b* = 10.044 (2) Å
*c* = 13.650 (3) Å
β = 110.749 (3)°
*V* = 2558.2 (10) Å^3^
*Z* = 4
Mo *K*α radiation
μ = 0.88 mm^−1^
*T* = 298 (2) K
0.41 × 0.31 × 0.16 mm

#### Data collection

Bruker SMART APEX CCD diffractometer
Absorption correction: multi-scan *SADABS* (Sheldrick, 1996 ▶) *T*_min_ = 0.714, *T*_max_ = 0.872
7123 measured reflections
2768 independent reflections
2361 reflections with *I* > 2σ(*I*)
*R*_int_ = 0.032

#### Refinement

*R*[*F* ^2^ > 2σ(*F* ^2^)] = 0.039
*wR*(*F* ^2^) = 0.108
*S* = 1.09
2768 reflections
168 parameters
1 restraint
H-atom parameters constrained
Δρ_max_ = 0.75 e Å^−3^
Δρ_min_ = −0.37 e Å^−3^

### 

Data collection: *SMART* (Bruker, 2007 ▶); cell refinement: *SAINT* (Bruker, 2007 ▶); data reduction: *SAINT*; program(s) used to solve structure: *SHELXTL* (Sheldrick, 2008 ▶); program(s) used to refine structure: *SHELXTL*; molecular graphics: *SHELXTL*; software used to prepare material for publication: *SHELXTL*.

## Supplementary Material

Crystal structure: contains datablocks I, global. DOI: 10.1107/S1600536808030882/lh2698sup1.cif

Structure factors: contains datablocks I. DOI: 10.1107/S1600536808030882/lh2698Isup2.hkl

Additional supplementary materials:  crystallographic information; 3D view; checkCIF report

## Figures and Tables

**Table:** 

Co1—N4	2.0597 (19)
Co1—O2	2.1394 (13)
Co1—N3	2.1856 (15)

**Table:** 

N4—Co1—N4^i^	175.43 (9)
N4—Co1—O2	91.49 (6)
N4^i^—Co1—O2	85.34 (6)
O2—Co1—O2^i^	92.24 (8)
N4—Co1—N3^i^	92.63 (6)
O2—Co1—N3^i^	173.74 (6)
N4—Co1—N3	90.81 (6)
O2—Co1—N3	92.79 (6)
N3^i^—Co1—N3	82.45 (8)

Symmetry code: (i) 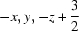.

**Table 2:** Hydrogen-bond geometry (Å, °)

*D*—H⋯*A*	*D*—H	H⋯*A*	*D*⋯*A*	*D*—H⋯*A*
O2—H5⋯S1^ii^	0.86	2.58	3.4123 (17)	164
O2—H8⋯N1^iii^	0.83	1.98	2.803 (2)	172

Symmetry codes: (ii) 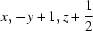; (iii) 
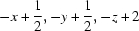
.

**Fig. 1.**
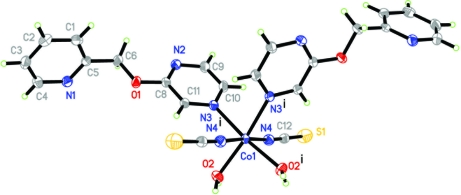
View of complex (I), showing the the asymmetric atom numbering scheme with thermal ellipsoids drawn at the 30% probability level. (symmetry code: (i) -x, y, -z + 3/2)

**Fig. 2.**
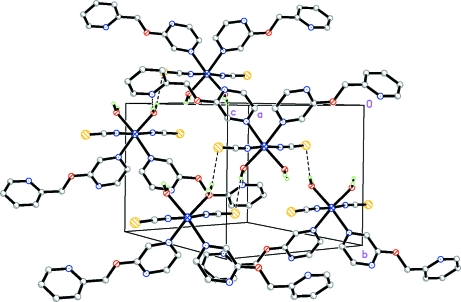
Part of the crystal structure of (I). Hydrogen bonds are shown as dashed lines.

## supplementary crystallographic information

### Comment

Molecules containing both pyridyl and pyrazinyl groups are useful multi-dentate ligands and a number of complexes have been published dealing with these ligands (e.g. Zhao *et al.*, 2007; Li, 2007). Herein the crystal structure of the title complex, (I), with 2-((pyridin-2-yl)methoxy)pyrazine as ligand, is reported.

The molecular structure of (I) is shown in Fig. 1. In the mono-nuclear complex, atom Co1 lies on a twofold rotation axis and its coordination geometry is slightly distorted octahedral (Table 1). In the crystal structure, complex molecules are linked via intermolecular O—H···S and O—H···N hydrogen bonds as shown in Fig. 2 and Table 2, to form a three-dimensional network. The diheral angle between pyridine and pyrazine rings is 85.86 (10)°. The title compound is isostructural with the Mn^II^ complex (Li, 2007).

### Experimental

A 5 ml methanol solution of 2-[(pyridin-2-yl)methoxy]pyrazine (0.0526 g, 0.281 mmol) was added into 10 ml H_2_O solution containing Co(ClO_4_)_2_.6H_2_O (0.1032 g, 0.282 mmol) and NaSCN (0.0457 g, 0.564 mmol), and the mixture was stirred for a few minutes. Red single crystals were obtained after the solution had been allowed to stand at room temperature for two weeks.

### Refinement

The H atoms bonded to O atoms were located in a difference Fourier map, and included in their 'as found' positions. The C-bound H atoms were placed in calculated positions, C—H = 0.93–0.97 Å. All H atoms were refined as riding, with *U*_iso_(H) = 1.2–1.5*U*_eq_(C,*O*).

### Crystal data

**Table:**

[Co(NCS)_2_(C_10_H_9_N_3_O)_2_(H_2_O)_2_]	*F*(000) = 1204
*M**_r_* = 585.53	*D*_x_ = 1.520 Mg m^−^^3^
Monoclinic, *C*2/*c*	Mo *K*α radiation, λ = 0.71073 Å
Hall symbol: -C 2yc	Cell parameters from 3335 reflections
*a* = 19.954 (4) Å	θ = 2.3–27.5°
*b* = 10.044 (2) Å	µ = 0.88 mm^−^^1^
*c* = 13.650 (3) Å	*T* = 298 K
β = 110.749 (3)°	Block, red
*V* = 2558.2 (10) Å^3^	0.41 × 0.31 × 0.16 mm
*Z* = 4	

### Data collection

**Table:**

Bruker SMART APEX CCD diffractometer	2768 independent reflections
Radiation source: fine-focus sealed tube	2361 reflections with *I* > 2σ(*I*)
graphite	*R*_int_ = 0.032
φ and ω scans	θ_max_ = 27.0°, θ_min_ = 2.2°
Absorption correction: multi-scan SADABS (Sheldrick, 1996)	*h* = −25→21
*T*_min_ = 0.714, *T*_max_ = 0.872	*k* = −11→12
7123 measured reflections	*l* = −9→17

### Refinement

**Table:**

Refinement on *F*^2^	Primary atom site location: structure-invariant direct methods
Least-squares matrix: full	Secondary atom site location: difference Fourier map
*R*[*F*^2^ > 2σ(*F*^2^)] = 0.039	Hydrogen site location: inferred from neighbouring sites
*wR*(*F*^2^) = 0.108	H-atom parameters constrained
*S* = 1.09	*w* = 1/[σ^2^(*F*_o_^2^) + (0.0638*P*)^2^ + 0.3122*P*] where *P* = (*F*_o_^2^ + 2*F*_c_^2^)/3
2768 reflections	(Δ/σ)_max_ = 0.008
168 parameters	Δρ_max_ = 0.75 e Å^−^^3^
1 restraint	Δρ_min_ = −0.37 e Å^−^^3^

### Special details

**Table:**

Geometry. All e.s.d.'s (except the e.s.d. in the dihedral angle between two l.s. planes) are estimated using the full covariance matrix. The cell e.s.d.'s are taken into account individually in the estimation of e.s.d.'s in distances, angles and torsion angles; correlations between e.s.d.'s in cell parameters are only used when they are defined by crystal symmetry. An approximate (isotropic) treatment of cell e.s.d.'s is used for estimating e.s.d.'s involving l.s. planes.
Refinement. Refinement of *F*^2^ against ALL reflections. The weighted *R*-factor *wR* and goodness of fit *S* are based on *F*^2^, conventional *R*-factors *R* are based on *F*, with *F* set to zero for negative *F*^2^. The threshold expression of *F*^2^ > σ(*F*^2^) is used only for calculating *R*-factors(gt) *etc*. and is not relevant to the choice of reflections for refinement. *R*-factors based on *F*^2^ are statistically about twice as large as those based on *F*, and *R*- factors based on ALL data will be even larger.

### Fractional atomic coordinates and isotropic or equivalent isotropic displacement parameters (Å^2^)

**Table:**

	*x*	*y*	*z*	*U*_iso_*/*U*_eq_	
C1	0.23226 (11)	−0.20768 (19)	1.25209 (19)	0.0415 (5)	
H1	0.1927	−0.2583	1.2136	0.050*	
C2	0.25719 (13)	−0.2109 (2)	1.3601 (2)	0.0473 (5)	
H2	0.2351	−0.2643	1.3955	0.057*	
C3	0.31522 (12)	−0.1337 (2)	1.41439 (17)	0.0471 (5)	
H3	0.3328	−0.1326	1.4872	0.057*	
C4	0.34682 (12)	−0.0580 (2)	1.35887 (17)	0.0474 (5)	
H4	0.3863	−0.0062	1.3961	0.057*	
C5	0.26655 (10)	−0.12879 (17)	1.20171 (15)	0.0336 (4)	
C6	0.24197 (11)	−0.1258 (2)	1.08505 (16)	0.0431 (5)	
H6A	0.2825	−0.1138	1.0625	0.052*	
H6B	0.2184	−0.2089	1.0562	0.052*	
N2	0.17307 (9)	−0.09050 (15)	0.88100 (13)	0.0385 (4)	
C8	0.15889 (10)	−0.00579 (18)	0.94509 (15)	0.0349 (4)	
C9	0.13592 (11)	−0.0711 (2)	0.77904 (16)	0.0425 (5)	
H9	0.1426	−0.1301	0.7308	0.051*	
C10	0.08838 (10)	0.0321 (2)	0.74250 (16)	0.0398 (5)	
H10	0.0646	0.0421	0.6708	0.048*	
C11	0.10991 (10)	0.09863 (18)	0.91016 (15)	0.0341 (4)	
H11	0.1012	0.1545	0.9587	0.041*	
C12	0.02714 (11)	0.28829 (18)	0.53206 (17)	0.0365 (4)	
Co1	0.0000	0.28266 (3)	0.7500	0.02902 (14)	
N4	0.02348 (10)	0.29084 (16)	0.61484 (15)	0.0394 (4)	
N3	0.07579 (8)	0.11899 (15)	0.80922 (13)	0.0330 (3)	
N1	0.32395 (9)	−0.05466 (17)	1.25423 (13)	0.0402 (4)	
O1	0.19253 (8)	−0.01598 (14)	1.04945 (11)	0.0455 (4)	
O2	0.07922 (7)	0.43031 (14)	0.82323 (11)	0.0425 (3)	
H5	0.0637	0.4916	0.8532	0.064*	
H8	0.1079	0.4599	0.7969	0.064*	
S1	0.03352 (5)	0.28165 (7)	0.41650 (6)	0.0722 (2)	

### Atomic displacement parameters (Å^2^)

**Table:**

	*U*^11^	*U*^22^	*U*^33^	*U*^12^	*U*^13^	*U*^23^
C1	0.0319 (10)	0.0342 (10)	0.0538 (13)	0.0006 (7)	0.0096 (10)	0.0005 (9)
C2	0.0484 (13)	0.0442 (11)	0.0552 (14)	0.0083 (9)	0.0257 (11)	0.0133 (10)
C3	0.0505 (13)	0.0511 (12)	0.0351 (11)	0.0126 (10)	0.0096 (10)	0.0053 (10)
C4	0.0409 (11)	0.0471 (12)	0.0439 (12)	−0.0040 (9)	0.0022 (10)	−0.0025 (10)
C5	0.0296 (9)	0.0283 (9)	0.0380 (10)	0.0092 (7)	0.0059 (8)	0.0013 (7)
C6	0.0425 (11)	0.0390 (10)	0.0387 (11)	0.0150 (9)	0.0032 (9)	0.0006 (9)
N2	0.0362 (9)	0.0342 (8)	0.0403 (9)	0.0070 (7)	0.0075 (7)	−0.0012 (7)
C8	0.0310 (9)	0.0330 (9)	0.0339 (10)	0.0013 (7)	0.0032 (8)	−0.0014 (8)
C9	0.0439 (11)	0.0420 (11)	0.0367 (11)	0.0094 (9)	0.0082 (9)	−0.0046 (9)
C10	0.0362 (10)	0.0425 (10)	0.0347 (10)	0.0066 (8)	0.0051 (9)	−0.0033 (9)
C11	0.0294 (9)	0.0315 (9)	0.0354 (10)	0.0028 (7)	0.0040 (8)	−0.0028 (8)
C12	0.0364 (10)	0.0331 (9)	0.0404 (9)	0.0018 (7)	0.0140 (9)	0.0011 (8)
Co1	0.0249 (2)	0.0302 (2)	0.0284 (2)	0.000	0.00494 (15)	0.000
N4	0.0405 (10)	0.0391 (9)	0.0397 (9)	−0.0016 (7)	0.0152 (8)	0.0003 (7)
N3	0.0257 (7)	0.0328 (8)	0.0354 (8)	0.0009 (6)	0.0045 (6)	−0.0013 (6)
N1	0.0346 (9)	0.0398 (9)	0.0419 (9)	−0.0016 (7)	0.0083 (8)	0.0039 (8)
O1	0.0514 (9)	0.0385 (7)	0.0343 (8)	0.0187 (6)	−0.0002 (7)	−0.0015 (6)
O2	0.0415 (8)	0.0437 (8)	0.0417 (8)	−0.0125 (6)	0.0137 (7)	−0.0072 (6)
S1	0.1124 (7)	0.0671 (5)	0.0530 (4)	0.0083 (4)	0.0491 (4)	0.0012 (3)

### Geometric parameters (Å, °)

**Table:**

C1—C5	1.379 (3)	C8—C11	1.397 (2)
C1—C2	1.380 (3)	C9—C10	1.373 (3)
C1—H1	0.9300	C9—H9	0.9300
C2—C3	1.372 (3)	C10—N3	1.348 (2)
C2—H2	0.9300	C10—H10	0.9300
C3—C4	1.374 (3)	C11—N3	1.319 (2)
C3—H3	0.9300	C11—H11	0.9300
C4—N1	1.337 (3)	C12—N4	1.158 (3)
C4—H4	0.9300	C12—S1	1.628 (2)
C5—N1	1.341 (2)	Co1—N4	2.0597 (19)
C5—C6	1.491 (3)	Co1—N4^i^	2.0597 (19)
C6—O1	1.445 (2)	Co1—O2	2.1394 (13)
C6—H6A	0.9700	Co1—O2^i^	2.1394 (13)
C6—H6B	0.9700	Co1—N3^i^	2.1856 (15)
N2—C8	1.321 (2)	Co1—N3	2.1856 (15)
N2—C9	1.339 (3)	O2—H5	0.8552
C8—O1	1.346 (2)	O2—H8	0.8316
			
C5—C1—C2	119.44 (19)	C9—C10—H10	119.6
C5—C1—H1	120.3	N3—C11—C8	120.83 (17)
C2—C1—H1	120.3	N3—C11—H11	119.6
C3—C2—C1	118.7 (2)	C8—C11—H11	119.6
C3—C2—H2	120.7	N4—C12—S1	178.7 (2)
C1—C2—H2	120.7	N4—Co1—N4^i^	175.43 (9)
C2—C3—C4	118.6 (2)	N4—Co1—O2	91.49 (6)
C2—C3—H3	120.7	N4^i^—Co1—O2	85.34 (6)
C4—C3—H3	120.7	N4—Co1—O2^i^	85.34 (6)
N1—C4—C3	123.60 (19)	N4^i^—Co1—O2^i^	91.49 (6)
N1—C4—H4	118.2	O2—Co1—O2^i^	92.24 (8)
C3—C4—H4	118.2	N4—Co1—N3^i^	92.63 (6)
N1—C5—C1	122.21 (19)	N4^i^—Co1—N3^i^	90.81 (6)
N1—C5—C6	117.08 (18)	O2—Co1—N3^i^	173.74 (6)
C1—C5—C6	120.69 (18)	O2^i^—Co1—N3^i^	92.79 (6)
O1—C6—C5	107.55 (16)	N4—Co1—N3	90.81 (6)
O1—C6—H6A	110.2	N4^i^—Co1—N3	92.63 (6)
C5—C6—H6A	110.2	O2—Co1—N3	92.79 (6)
O1—C6—H6B	110.2	O2^i^—Co1—N3	173.74 (6)
C5—C6—H6B	110.2	N3^i^—Co1—N3	82.45 (8)
H6A—C6—H6B	108.5	C12—N4—Co1	170.41 (18)
C8—N2—C9	115.14 (16)	C11—N3—C10	117.06 (16)
N2—C8—O1	120.55 (16)	C11—N3—Co1	122.44 (12)
N2—C8—C11	123.07 (17)	C10—N3—Co1	120.49 (13)
O1—C8—C11	116.38 (17)	C4—N1—C5	117.44 (17)
N2—C9—C10	122.97 (19)	C8—O1—C6	116.04 (15)
N2—C9—H9	118.5	Co1—O2—H5	113.0
C10—C9—H9	118.5	Co1—O2—H8	123.6
N3—C10—C9	120.87 (19)	H5—O2—H8	111.7
N3—C10—H10	119.6		
			
C5—C1—C2—C3	0.8 (3)	C9—C10—N3—Co1	180.00 (15)
C1—C2—C3—C4	−1.0 (3)	N4—Co1—N3—C11	150.92 (14)
C2—C3—C4—N1	0.3 (3)	N4^i^—Co1—N3—C11	−26.06 (15)
C2—C1—C5—N1	0.3 (3)	O2—Co1—N3—C11	59.39 (14)
C2—C1—C5—C6	178.48 (17)	N3^i^—Co1—N3—C11	−116.53 (16)
N1—C5—C6—O1	−88.0 (2)	N4—Co1—N3—C10	−30.49 (15)
C1—C5—C6—O1	93.7 (2)	N4^i^—Co1—N3—C10	152.53 (15)
C9—N2—C8—O1	179.51 (18)	O2—Co1—N3—C10	−122.02 (14)
C9—N2—C8—C11	−1.2 (3)	N3^i^—Co1—N3—C10	62.06 (13)
C8—N2—C9—C10	2.4 (3)	C3—C4—N1—C5	0.7 (3)
N2—C9—C10—N3	−1.2 (3)	C1—C5—N1—C4	−1.0 (3)
N2—C8—C11—N3	−1.2 (3)	C6—C5—N1—C4	−179.25 (17)
O1—C8—C11—N3	178.10 (17)	N2—C8—O1—C6	−1.9 (3)
C8—C11—N3—C10	2.4 (3)	C11—C8—O1—C6	178.76 (17)
C8—C11—N3—Co1	−178.94 (13)	C5—C6—O1—C8	−173.42 (17)
C9—C10—N3—C11	−1.3 (3)		

Symmetry codes: (i) −*x*, *y*, −*z*+3/2.

### Hydrogen-bond geometry (Å, °)

**Table:**

*D*—H···*A*	*D*—H	H···*A*	*D*···*A*	*D*—H···*A*
O2—H5···S1^ii^	0.86	2.58	3.4123 (17)	164.
O2—H8···N1^iii^	0.83	1.98	2.803 (2)	172.

Symmetry codes: (ii) *x*, −*y*+1, *z*+1/2; (iii) −*x*+1/2, −*y*+1/2, −*z*+2.
